# Synergistic Catalysis of Gold–Platinum Alloy Nanozymes: A Novel Colorimetric Sensor for ALP Detection in Complex Biological Matrices

**DOI:** 10.3390/ph18121795

**Published:** 2025-11-25

**Authors:** Baojie Du, Bingqing Zhang, Xiaofeng Ren, Jie Yang, Fan Yang, Chunyu Yan, Liping Li, Ruiping Zhang

**Affiliations:** 1 Shanxi Bethune Hospital, Shanxi Academy of Medical Sciences, Third Hospital of Shanxi Medical University, Tongji Shanxi Hospital, Taiyuan 030032, China; dubaojie@sxbqeh.com.cn (B.D.); yangjie@sxbqeh.com.cn (J.Y.); 2 Department of Biochemistry and Molecular Biology, School of Basic Medical Sciences, Shanxi Medical University, Taiyuan 030001, China; b2024035015@student.pumc.edu.cn (B.Z.); dr20240041087@sxmu.edu.cn (X.R.); yangfan12@sxmu.edu.cn (F.Y.); yanchunyu@sxmu.edu.cn (C.Y.); 3 Radiology Department of Shanxi Provincial People’s Hospital, Five Hospital of Shanxi Medical University, Taiyuan 030001, China

**Keywords:** bimetallic nanozyme, colorimetric detection, alkaline phosphatase, sensor

## Abstract

**Background/Objectives**: Alkaline phosphatase (ALP) is a crucial enzyme in numerous pathological processes and a significant biomarker in clinical diagnostics. Conventional ALP detection methods are hampered by reliance on complex sample pretreatment, sophisticated instrumentation, time-consuming procedures, and high costs. This study aimed to develop a simple, rapid, and cost-effective colorimetric sensing method for ALP detection with enhanced resistance to matrix interference in biological samples. **Methods**: We designed a colorimetric assay based on bimetallic gold–platinum nanocatalysts (AuPt NPs) exhibiting peroxidase-like (POD-like) activity. The detection principle involves a dual-reaction cascade: (1) Alkaline phosphatase (ALP) catalyzes the conversion of trisodium L-ascorbic acid-2-phosphate (AA_2_P) into ascorbic acid (AA), and (2) the generated AA reduces oxidized 3,3′,5,5′-tetramethylbenzidine (oxTMB) produced by the catalytic activity of AuPt NPs. This method was evaluated for its detection performance in diluted human serum without complex sample pretreatment. **Results**: AuPt NPs exhibited resistance to biological matrix interference, enabling sensitive detection of ALP. The assay showed a linear ALP detection range of 0–90 mU·mL^−1^ (R^2^ = 0.994) and a limit of detection of 3.91 mU·mL^−1^. In spiked human serum, recoveries were 95.45–111.97%, with negligible interference from ions and biomolecules. **Conclusions**: We developed a simple, rapid, and reliable colorimetric sensor for ALP detection based on AuPt NPs. It overcomes limitations of conventional methods, holding great potential for clinical diagnostics and point-of-care applications.

## 1. Introduction

ALP activity in biological systems demonstrates strong correlations with the pathophysiological mechanisms of diverse diseases, including metabolic disease [1], bone injury [2], cancer [3] and other diseases [4,5,6]. Therefore, efficient and sensitive detection of ALP activity levels in clinical specimens is essential for early diagnosis and effective treatment. Conventional ALP analyzing techniques—surface plasmon resonance biosensors [4,5], electrochemical detection methods [7], fluorescent detection methods [8,9], and electrochemical impedance methods—rely on preprocessing steps to mitigate biomatrix effects, compromising analytical simplicity and point-of-care applicability [10,11]. Further, these techniques are limited by insufficient sensitivity, operational complexity, and expensive instrumentation dependency. The development of simple, efficient, and sensitive ALP detection methodologies remains a focal area of contemporary research.

Colorimetric assays, predicated on chromogenic reactions, leverage highly sensitive and broadly applicable chromogenic substrates as critical determinants for innovative method development [3]. In addition, they offer a promising alternative due to rapid visual readout and minimal instrumentation [12]. However, existing chromogenic platforms exhibit insufficient tolerance to biological interferents (e.g., proteins, bilirubin), limiting direct analysis of crude samples [13]. There is an urgent need to develop novel materials that can significantly enhance chromogenic reaction sensitivity and broaden the detection range in colorimetric assays.

To overcome this barrier, nanozyme-based sensor assays have emerged, leveraging catalytic nanomaterials to enhance signal robustness in complex matrices. Furthermore, nanozymes also provide synthetic accessibility, exceptional catalytic versatility, and operational robustness for ALP detection [14,15]. Hsieh et al. demonstrated the utility of carbon-based nanostructured active sites in ALP activity colorimetric assays, investigating nanozyme recognition/binding capabilities and confirming the selective sensitivity of non-metallic nanozyme systems [16]. Song et al. employed a manganese-based metal–organic gel nanozyme rich in oxygen vacancies to measure ALP activity, revealing its notable oxidase-mimicking activity [17]. Tan et al. developed hollow AuAg@CeO_2_ plasmonic nanozymes for ALP detection via the triple synergistic effects of hot electron injection, photothermal activation, and localized surface plasmon resonance (LSPR), demonstrating enhanced peroxidase-like activity, 100-fold sensitivity improvement over commercial kits, 10 min rapid assay, and high anti-interference ability with 94.8–105.4% recovery in clinical samples [18]. The diversity of nanozymes has been extensively explored, revealing significant variations in catalytic activity among nanozymes derived from different material sources. Therefore, systematic exploration of nanozyme systems with improved catalytic activity is imperative for advancing and efficient biomedical applications. Noble metals-based nanozymes demonstrate significant potential in biomedical applications due to their distinctive surface effects and optoelectronic properties [19]. Nevertheless, most reported nanozymes still require preprocessing or lack validated anti-interference capability in clinical samples. Compared to monometallic nanozymes [14], bimetallic nanozymes exhibit superior catalytic performance arising from synergistic interaction between the two metals [20,21]. Bimetallic nanozymes exhibit remarkable advantages in enzyme detection by virtue of their unique physicochemical properties. In terms of sample pretreatment, the rich active sites and tunable surface properties of bimetallic nanozymes enable efficient enrichment and purification of target enzymes from complex samples through specific adsorption or catalytic reactions, thereby reducing the tedious procedures of traditional pretreatment. Meanwhile, the synergistic effect of bimetallic components endows them with specific electronic structures and catalytic activities, allowing target enzymes to be recognized and catalyzed based on structure-function relationships. This mechanism effectively resists interferences from coexisting substances in complex matrices. Combined with their tunable optical and electrical properties, bimetallic nanozymes can construct highly sensitive detection systems, providing efficient and accurate solutions for enzyme detection in complex environments.

**Scheme 1:**
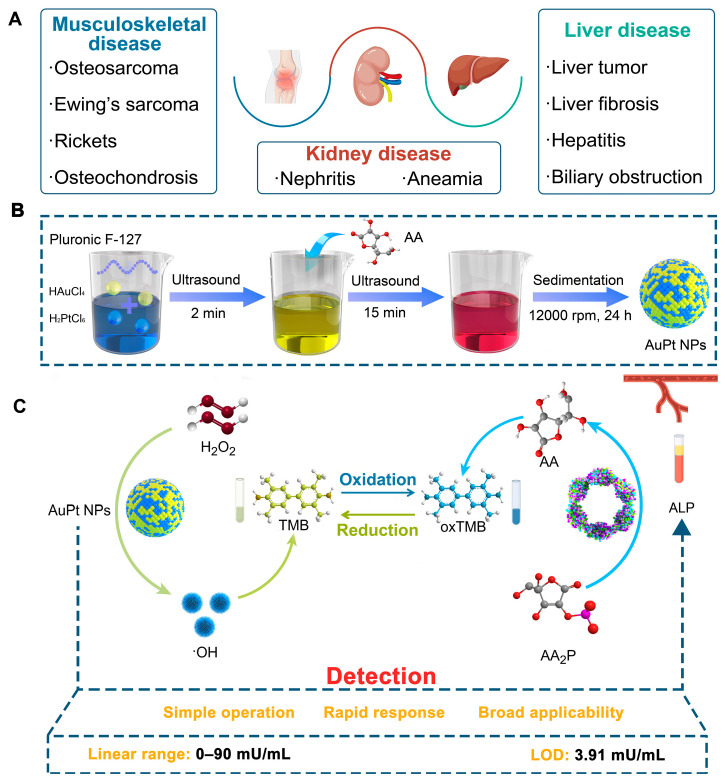
Schematic illustration of colorimetric detection method for ALP based on AuPt NPs. (**A**) The clinical significance of detecting ALP; (**B**) Rapid preparation method for AuPt NPs catalyzing the color change of chromogenic agents; (**C**) ALP colorimetric analysis based on AuPt NPs.

Herein, we develop a colorimetric biosensing assay, exploiting the intrinsic matrix resistance of POD-like activity of AuPt bimetallic alloy nanozymes. This strategy enables direct ALP detection in human serum through a cascade reaction: in the presence of H_2_O_2_, AuPt nanozymes are activated to catalyze the generation of hydroxyl radicals (·OH), which mediate the oxidation of colorless 3,3′,5,5′-tetramethylbenzidine (TMB) to blue oxTMB, imparting a blue color to the solution. When ALP is present, it specifically catalyzes the dephosphorylation of AA_2_P, releasing AA. As a reducing agent, AA competitively reduces oxTMB back to TMB, leading to the color transition of the solution from blue to colorless. The degree of color change in the system correlates directly with the concentration of ALP. Higher ALP concentrations result in more AA production, greater reduction of oxTMB, and more pronounced color fading, enabling quantitative analysis via visual observation or absorbance measurement. Furthermore, the TMB-H_2_O_2_-AuPt nanozyme system exhibits high catalytic efficiency, excellent operational stability, and good reproducibility across multiple trials, providing a simple and robust strategy for ALP detection. The high chemical stability of AuPt NPs endows them with a robust surface that resists degradation by biomolecular contaminants. Additionally, the bimetallic active sites exhibit enhanced catalytic efficiency toward target substrates while suppressing side reactions with interfering species, ensuring selective recognition. The AuPt NPs’ surface properties confer exceptional stability against biomolecular fouling, eliminating complex preprocessing while maintaining >95 recovery in spiked clinical specimens. This approach provides a rapid, interference-resistant assay for ALP quantification, advancing robust clinical analysis (Scheme 1).

## 2. Results

### 2.1. Characterization of AuPt NPs Catalyzing the Color Reaction of Chromogenic Agents

**Figure 1:**
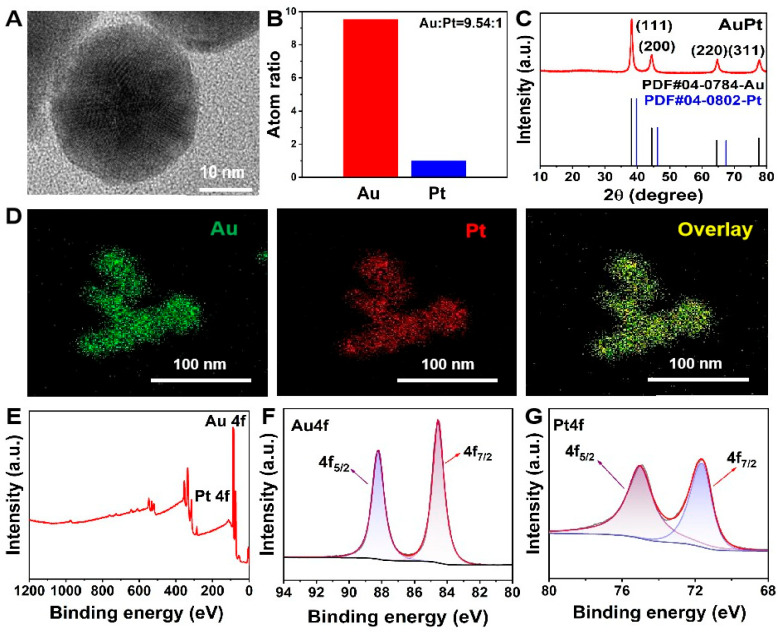
Characterizations of AuPt NPs. (**A**) TEM image of AuPt NPs; (**B**) Quantitative analysis of the elemental composition of AuPt NPs using ICP-MS; (**C**) XRD pattern of AuPt NPs; (**D**) EDS elemental mapping to confirm the coexistence of Au and Pt in AuPt NPs; (**E**) XPS pattern of AuPt NPs; (**F**) High-resolution XPS spectra of Au 4f in AuPt NPs; (**G**) High-resolution XPS spectra of Pt 4f in AuPt NPs.

To enhance the sensitivity of colorimetric detection for ALP, we prepared AuPt NPs. The structural properties of the synthesized nanoparticles were exhibited by scanning electron microscopy (SEM) and transmission electron microscopy (TEM). The results showed that AuPt NPs possess a well-defined spherical morphology with an average diameter of 20 nm (Figure 1A and Figure S1A–C), which is optimal for catalytic applications owing to the high surface-to-volume ratio. Inductively coupled plasma mass spectrometry (ICP-MS) was used to verify the elemental composition, revealing a gold-to-platinum molar ratio of 9.54:1 (Figure 1B). The crystalline structure was determined by X-ray diffraction (XRD), with diffraction peaks observed at (111), (200), (220), and (311) planes, which match the standard face-centered cubic Au phase (JCPDS No. 04-0784; Figure 1C). Due to the low Pt content, the characteristic peaks corresponding to the Pt phase (JCPDS No. 04-0802) were less pronounced. Energy-dispersive X-ray spectroscopy (EDS) elemental mapping confirms the homogeneous distribution of Au and Pt within the nanoparticles (Figure 1D and Figure S1D). X-ray photoelectron spectroscopy (XPS) analysis further confirmed the metallic states of Au and Pt, with Au 4f peaks at 84.58 eV (Au 4f7/2) and 88.18 eV (Au 4f5/2), and Pt 4f peaks at 71.68 eV (Pt 4f7/2) and 74.88 eV (Pt 4f5/2) (Figure 1E–G).

### 2.2. The Colorimetric Detection of ALP Based on AuPt NPs

**Figure 2:**
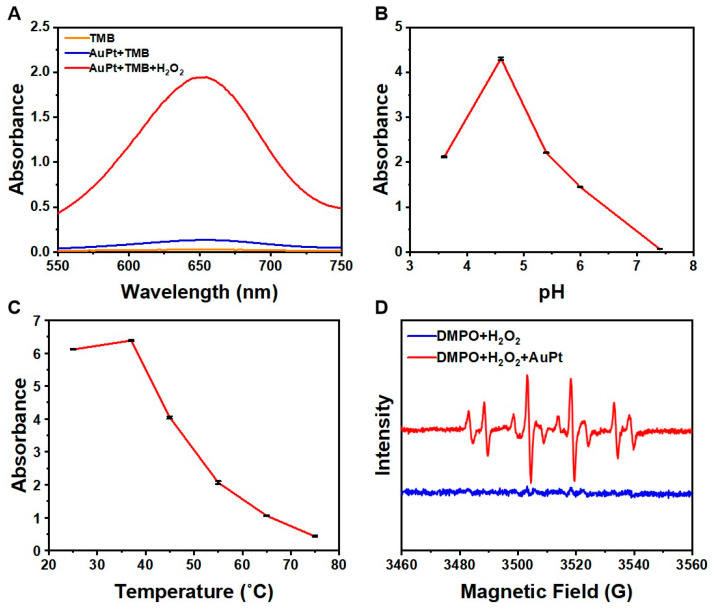
Enzymatic performance of AuPt NPs. (**A**) POD-like activity of AuPt NPs measured by a TMB probe; (**B**) Dependency of the POD-like activity on pH; (**C**) Dependency of the POD-like activity on temperature; (**D**) ESR spectra of DMPO/OH adducts. (Error bars: repeated experiments; *n* = 3).

To evaluate the application potential of AuPt NPs in colorimetric methods, we first assessed their POD-like catalytic performance. The reaction produced a characteristic blue color with a distinct absorbance peak at 652 nm (Figure 2A), demonstrating the intrinsic POD-like activity of the nanoparticles. Systematic optimization of the catalytic performance revealed pH- and temperature-dependent behaviors, with optimal performance observed at pH 4.6 and 37 °C (Figure 2B,C). The observed pH and temperature profiles suggest that the AuPt NPs exhibit robust catalytic performance, making them particularly suitable for diagnostic applications. To elucidate the underlying catalytic mechanism and better understand the structure-activity relationship, ESR spectroscopy was employed using 5,5-dimethyl-1-pyrroline N-oxide (DMPO) as a spin trap. The results reveal the generation of ·OH, as evidenced by the characteristic 1:2:2:1 quartet signal (Figure 2D).

### 2.3. Calculation of the Steady-State Kinetics

**Figure 3:**
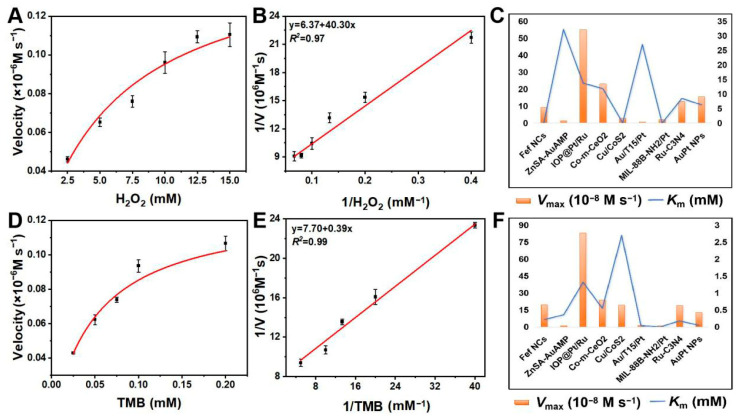
Steady-state kinetic assay of AuPt NPs. (**A**,**D**) Michaelis–Menten plots and (**B**,**E**) double reciprocal plots of AuPt NPs; (**C**,**F**) *K*m and *V*max for the reaction of different catalysts with substrates H_2_O_2_ and TMB. The concentration of TMB in (**A**) was 0.5 mM and the H_2_O_2_ concentration in (**D**) was 25 mM. (Error bars: repeated experiments; *n* = 3).

To evaluate the catalytic performance of AuPt NPs in the sensing method for ALP detection, systematic characterization was performed to assess their activity and selectivity in colorimetric assays. Comprehensive steady-state kinetic analyses were conducted using the Michaelis–Menten enzymatic model. As shown in the figure (Figure 3A,D), the reaction kinetics display characteristic saturation behavior, where the initial reaction velocities increased rapidly at low substrate concentrations before reaching plateaus at elevated H_2_O_2_ and TMB concentrations. The corresponding double reciprocal plots (Figure 3B,E) enabled precise determination of kinetic parameters, yielding a *K*m of 0.050 mM and *V*max of 12.79 × 10^−8^ M s^−1^ for TMB oxidation, along with a *K*m of 6.31 mM and *V*max of 15.54 × 10^−8^ M s^−1^ for H_2_O_2_ decomposition.

Through systematic kinetic characterization and comparative analysis with the recent nanozyme systems (Table S1), the AuPt NPs demonstrate superior catalytic performance that combines high substrate affinity with turnover rates, enabling rapid and specific biochemical transformations even at low concentrations. The *K*m of AuPt NPs for TMB oxidation indicates remarkably strong substrate binding affinity (Figure 3C), representing a 4.4-fold improvement over iron-based nanocatalysts (Fef NCs, 0.22 mM) and a 7.2-fold enhancement compared to zinc-stabilized gold systems (ZnSA-AuAMP hydrogel, 0.36 mM). Compared to AuPt NPs, only a small number of systems exhibit better performance in terms of substrate affinity (MIL-88B-NH_2_/Pt, 0.00213 mM). However, when considering the critical catalytic property of *V*max, the AuPt NPs developed in this study demonstrate more prominent advantages. For substrate H_2_O_2_, the AuPt NPs exhibit a *K*m of 6.31 mM coupled with a *V*max of 15.54 × 10^−8^ M s^−1^ (Figure 3F). These parameters position our system as significantly more efficient than conventional oxide-based nanozymes (Co-m-CeO_2_) and various metallic composites. The observed kinetic enhancement is attributed to bimetallic charge transfer, thereby boosting the nanozyme’s synergistic catalysis.

### 2.4. Inhibiting Effect of AA on POD-like Activity of AuPt NPs

**Figure 4:**
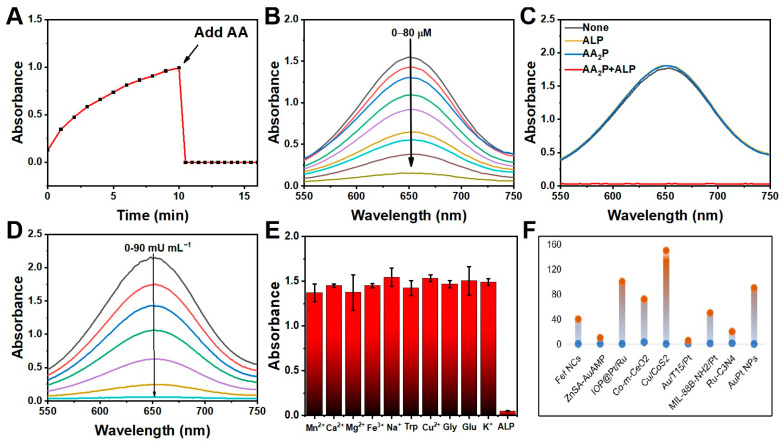
Colorimetric detection of AA based on the oxidase-like activity of AuPt NPs. (**A**) Time-dependent absorbance at 652 nm for AuPt NPs-catalyzed TMB oxidation and AA-mediated reducing of oxidized TMB; (**B**) UV-vis absorption spectra of the reaction solutions with various concentrations of AA; (**C**) UV-vis absorption spectra in the presence of no reactants, ALP, AA_2_P, and both ALP and AA_2_P; (**D**) UV-vis absorption spectra with different activities of ALP; (**E**) Selectivity test of ALP detection; (**F**) The linear ranges exhibited by different catalysts. (Error bars: repeated experiments; *n* = 3).

The inhibitory interaction between AA and the POD-like activity of AuPt nanoparticles was systematically investigated to establish a robust detection platform. As illustrated in Figure 4A, the introduction of AA into the TMB-H_2_O_2_ reaction system resulted in immediate attenuation of the characteristic absorption band at 652 nm, corresponding to the reduction of oxTMB by AA through electron transfer processes. This redox reaction follows a stoichiometric relationship where one mole of AA reduces two moles of oxTMB, explaining the rapid signal diminution observed.

The quantitative analysis revealed a precise concentration-dependent response (0–80 μM AA range) with excellent linear correlation (A652 nm = −0.019[AA] + 1.67, R^2^ = 0.997) as shown in Figure 4B and Figure S2A. The achieved detection limit of 3.04 μM (S/N = 3) represents significant improvement over conventional AA detection methods, attributable to three key factors: (1) the exceptional catalytic activity of AuPt NPs ensures high initial oxTMB generation, (2) the strong reducing capability of AA enables complete oxTMB reduction, and (3) the distinct color transition provides optical signal output. The remarkable selectivity demonstrated in the Figure S2C stems from fundamental chemical principles. While common metal ions (Ca^2+^, Mn^2+^, K^+^, Na^+^, Cu^2+^, Mg^2+^) and biomolecules (glucose, glycine) showed negligible interference (<5% signal variation), AA exhibited >90% signal suppression.

### 2.5. ALP Quantification in Clinical Serum Samples

We have developed an innovative colorimetric assay for ALP detection by synergistically combining the POD-like activity of AuPt NPs with the enzymatic conversion of AA_2_P to AA. The system’s operational principle has been rigorously validated through controlled experiments, which demonstrate specific response to ALP activity, as indicated by significant signal suppression (*p* < 0.01) exclusively in samples containing both AA**_2_**P and ALP (Figure 4C).

Quantitative performance evaluation reveals an excellent linear correlation between the absorbance at 652 nm and ALP concentrations across the clinically relevant range of 0–90 mU mL^−1^ (Figure 4D). The regression equation A652 nm = −0.024 [ALP] + 2.11 (R^2^ = 0.994, *n* = 3) demonstrates remarkable linearity, with the near-unity coefficient of determination (R^2^) indicating that 99.4% of the absorbance variation can be accounted for by ALP concentration changes (Figure S2B). Such a strong linear relationship (*p* < 0.001) ensures reliable quantitative analysis across the entire detection range. The achieved detection limit of 3.91 mU mL^−1^ (S/N = 3) represents a 3–5-fold improvement over conventional ALP detection methods, enabling sensitive measurement of ALP activity at sub-pathological levels (Table 1).

The assay’s exceptional selectivity has been confirmed through comprehensive interference testing against major serum components, including divalent cations (Mn^2+^, Ca^2+^, Mg^2+^), transition metals (Fe^3+^, Cu^2+^), monovalent ions (Na^+^, K^+^), and biomolecules (glucose, glycine, tryptophan). As shown in Figure 4E, all tested interferents produce signal variations below 6.5% of the ALP response (*p* > 0.05), demonstrating negligible cross-reactivity. Comparative analysis of the catalytic performance reveals distinct advantages of AuPt NPs over existing systems (Figure 4F and Table 1). While exhibiting a comparable detection limit (3.91 mU mL^−1^) to most reference materials, the AuPt nanozymes demonstrate a broad linear range (0–90 mU mL^−1^). This balanced performance stems from the unique AuPt alloy structure, characterized by elemental distribution (EDS mapping) and electronic interaction (XPS-verified charge transfer), which enables simultaneous achievement of high substrate affinity (*K*m(TMB) = 0.050 mM) and rapid catalytic turnover (*V*max = 12.79 × 10^−8^ M s^−1^). Unlike complex composites requiring precise oxygen vacancy engineering (e.g., Co-m-CeO_2_) or specialized supports (e.g., MIL-88B-NH_2_/Pt), the AuPt system maintains robust activity through intrinsic metallic bonding (XRD), offering both superior operational stability and simpler synthesis than most compared nanozymes.

Clinical validation studies using spiked serum samples yield recovery rates of 95.45–111.97% (Table 2), with relative standard deviations (RSD) ranging from 0.71% to 10.98%. These results not only confirm the method’s accuracy in complex biological matrices but also suggest its potential for direct clinical application without extensive sample pretreatment.

## 3. Discussion

The characterization results of AuPt NPs collectively demonstrate the successful reduction of AuCl_4_^−^ and PtCl_6_^2−^ precursors to Au^0^ and Pt^0^, confirming the formation of AuPt alloys with potential catalytic applications [30]. The AuPt NPs exhibit a characteristic blue color with a distinct absorption peak at 652 nm, indicating that the NPs possess inherent pod-like activity. This property significantly reduces interference from the absorption peak of bilirubin in serum, which is centered at 405 nm. Moreover, compared with monometallic gold and platinum, as well as nanoclusters, the catalytic activity of alloy nanomaterials for oxidizing TMB in the presence of H_2_O_2_ is significantly enhanced [31]. This excellent POD-like activity, stemming from the synergistic electronic effects between Au and Pt in the alloy structure, lays a solid foundation for constructing sensitive biosensing platforms. This finding not only confirms that the POD-like activity of AuPt NPs follows a radical-mediated pathway similar to natural peroxidases, but also provides mechanistic insights into how the unique electronic structure of the AuPt alloy (as demonstrated by XRD and XPS analyses) contributes to its exceptional catalytic performance. The radical-mediated mechanism is particularly crucial for their application in oxidative catalysis and explains the observed high sensitivity in subsequent biosensing applications.

The exceptional kinetic parameters can be attributed to the unique structural advantages of the AuPt NPs. The homogeneous bimetallic composition (as confirmed by EDS mapping and XPS analysis) facilitates optimal electronic interaction between Au and Pt atoms, creating favorable electronic environments for substrate activation. The 9.54:1 Au:Pt molar ratio is critical in this context, as it balances active site accessibility and structural stability, maximizing the exposure of reactive sites while suppressing Pt self-aggregation that would otherwise diminish catalytic performance [32,33]. These intrinsic advantages rationalize why the as-synthesized AuPt NPs surpass both monometallic nanocatalysts and many reported bimetallic systems in peroxidase-mimicking catalysis, highlighting the synergy between compositional design and catalytic functionality [34,35].

The excellent selectivity of this detection platform enables it to achieve selective detection of AA even in complex matrices containing potential interferents at physiological concentrations. The visual color transition from intense blue to colorless provides additional practical advantages. Such optical signal amplification, coupled with the catalytic signal generation from AuPt NPs, explains the system’s superior sensitivity compared to direct AA detection methods.

This prominent specificity stems from two key molecular-level advantages: the enzyme-substrate specificity of ALP for AA_2_P, and the selective inhibition of AuPt NPs activity by enzymatically generated AA. The phosphophenyl diphosphate colorimetric method commonly used in clinical laboratories for determining alkaline phosphatase (ALP) activity has inherent technical limitations. This method is based on the hydrolysis of disodium phenyl phosphate by alkaline phosphatase to produce phenol and phosphate, and the color reaction of phenol with potassium ferricyanide for colorimetric determination. Its detection principle determines that it faces multiple challenges in practical applications: on the one hand, due to the lack of specificity of the color reaction, the spontaneous oxidation of bilirubin under alkaline conditions and the light absorption interference of hemoglobin released by hemolysis lead to deviations in absorbance measurement; on the other hand, this method requires multiple steps of manual operation (such as sample pretreatment, control of reagent addition order, strict control of reaction time, etc.), and any minor error in the operation process can accumulate into significant systematic errors, thereby affecting the accuracy of detection. In contrast, our method is more sensitive and reliable, simple to operate, and has high anti-interference ability.

Furthermore, the excellent clinical validation results not only confirm the accuracy of this method in complex biological matrices, but also demonstrate its potential for direct clinical application. The platform’s combined advantages of high sensitivity (enabled by AuPt NPs’ superior catalytic activity) and operational simplicity (colorimetric readout) position it as an ideal solution for point-of-care ALP testing in both diagnostic and research settings.

## 4. Materials and Methods

### 4.1. Materials

Chloroplatinic acid hexahydrate (H_2_PtCl_6_·6H_2_O), glacial acetic acid (C_2_H_4_O_2_), and tetrachloroauric acid trihydrate (HAuCl_4_·3H_2_O) were acquired from Aladdin (Shanghai, China). L-Tryptophan (C_11_H_12_N_2_O_2_) and ALP were purchased from Macklin. AA_2_P was bought from Sigma-Aldrich (Shanghai, China). AA is a product of Energy Chemical. Serum specimens were acquired from Shanxi Bethune Hospital, Taiyuan, China.

### 4.2. Synthesis of AuPt NPs

The synthesis of gold–platinum nanozymes was improved based on the existing methods [31,35]. The AuPt nanostructure was synthesized through a controlled reduction process, where 250 μL of 0.2 M HAuCl_4_·3H_2_O, 5 μL of H_2_PtCl_6_·6H_2_O (1 M), and 60 mg of Pluronic F-127 were initially dissolved in 2 mL deionized water under 2 min sonication. Following this, 10 mL of AA (30 mg mL^−1^) was rapidly introduced into the mixture, inducing an immediate color transition to wine red. The reaction system underwent additional sonication for 15 min before being maintained at ambient temperature for 24 h to complete the nanostructure formation. The resulting AuPt NPs were isolated through three cycles of centrifugation (12,000 rpm, 5 min) and subsequently redispersed in deionized water for further use.

### 4.3. Construction and Optimization of the Colorimetric System

The key to colorimetric detection resides in the highly efficient catalysis of chromogenic agents’ colorimetric reaction via the peroxidase-like activity of AuPt NPs. The POD-like activity was evaluated using TMB as the colorimetric substrate. In brief, 20 μL of a 1 mg mL^−1^ AuPt NPs solution was combined with 10 μL of TMB (100 mM), 5 μL of H_2_O_2_ (10 M), and 1965 μL of HAc-NaAc buffer (0.2 M, pH 4.6). After 10 min of incubation at room temperature, absorbance was measured using a UV-Vis spectrophotometer, Agilent Technologies, Beijing, China. The catalytic generation of ·OH from H_2_O_2_ by AuPt NPs was detected by electron spin resonance (ESR). Furthermore, to assess the effects of temperature and pH on the catalytic efficiency of AuPt NPs, the tests were performed under varying conditions, with temperatures set at 25, 37, 45, 55, 65, and 75 °C and pH values adjusted to 3.6, 4.6, 5.4, 6, and 7.6, following the same experimental procedures described above.

### 4.4. Steady-State Kinetic Analysis of AuPt NPs in Colorimetric Systems

To demonstrate the substrate affinity and reactivity of the AuPt NPs-based colorimetric assay for alkaline phosphatase (ALP) in clinical samples detection, we performed the kinetic characterization of AuPt NPs via steady-state analysis using the Michaelis–Menten model. The Michaelis–Menten equation is expressed mathematically as 1/*V* = (*K*m/*V*max) × (1/[S]) + 1/*V*max, where *V* indicates initial velocity, *V*max represents maximum velocity, [S] signifies substrate concentration, and *K*m stands for the Michaelis constant. The parameter *K*m, defined as the substrate concentration yielding half-maximal velocity, provides a quantitative measure of enzyme-substrate binding affinity. Standard experimental protocols were implemented to determine these kinetic parameters for the AuPt NP-catalyzed reactions.

The kinetic studies were systematically conducted using two distinct experimental setups. For H_2_O_2_ kinetics, reaction mixtures were prepared by combining 10 μL of TMB (100 mM), 20 μL of AuPt NPs (1 mg mL^−1^), and incremental additions of 10 M H_2_O_2_ (0–2.5 μL) in HAc-NaAc buffer (0.2 M, pH 4.6), with total volumes precisely adjusted to 2 mL through corresponding buffer volume modifications (1970–1967.5 μL). Absorbance measurements at 652 nm were acquired at 5 s intervals during the initial 180 s period. Parallel experiments examining TMB kinetics employed identical analytical conditions, but with varying TMB concentrations (0–0.2 mM) while maintaining fixed quantities of 5 μL 10 M H_2_O_2_, 20 μL AuPt NPs, and buffer in a 2 mL final volume, followed by equivalent absorbance monitoring protocols.

### 4.5. Detection of the Reducing Property and Selectivity of ALP Catalytic Products

The inhibitory effect of AA on the TMB oxidation process was quantitatively detected through a colorimetric assay, demonstrating AA’s significant reducing capacity in this catalytic system. First, 20 μL of a 1 mg mL^−1^ AuPt NPs was stirred into a solution containing 10 μL of TMB (100 mM), 5 μL of H_2_O_2_ (10 M) and 1945 μL HAc-NaAc buffer (0.2 M, pH 4.6). Different volumes (0, 10, 20, 30, 40, 50, 60, 70, 80 μL) of AA (10 mM) were added to the above solution and incubated for 10 min before detecting the absorbance at 652 nm. The selectivity of AA was assayed under the same conditions using a number of possible interferents, including Ca^2+^, Mn^2+^, K^+^, Na^+^, Cu^2+^, Mg^2+^, Glutamic acid (Glu), and Glycine (Gly).

### 4.6. Anti-Interference Detection of ALP Based on AuPt NPs

For ALP activity detection, 50 μL of 15 mM AA_2_P was diluted in 100 μL of Tris-HCl buffer (50 mM, pH 8.8) with 50 μL of ALP solutions at varying activities (0, 15, 30, 60, 70, 80, and 90 mU·mL^−1^). The mixture was incubated for 30 min at 37 °C. Subsequently, 100 μL of the reaction solution was pipetted into TMB-H_2_O_2_-AuPt NPs solution (25 mM H_2_O_2_, 10 μg·mL^−1^ AuPt NPs, 0.5 mM TMB) and incubation was continued for 10 min.

To evaluate selectivity, potential interferents—including Mn^2+^, Ca^2+^, Mg^2+^, Fe^3+^, Na^+^, Cu^2+^, K^+^, glutamic acid (Glu), glycine (Gly), and tryptophan (Trp)—were tested at a final concentration of 500 μM. Briefly, each interferent (replacing ALP) was added to a solution containing 3.75 mM AA_2_P in Tris-HCl buffer (25 mM, pH 8.8) and incubated for 30 min at 37 °C. Subsequent steps followed the same protocol as the ALP detection assay.

### 4.7. Assay of ALP in Serum Samples

Initially, serum samples were diluted 50-fold using a 50 mM Tris-HCl buffer (pH 8.8) (Aladdin) to control the ALP activity within the linear range of the detection system and avoid errors caused by exceeding the measurement range. Subsequently, diluted samples were spiked with ALP at different concentrations (10, 30, and 60 mU mL^−1^). The subsequent procedures followed those established for ALP detection. The serum sample collection was approved by the Ethics Committee of Shanxi Bethune Hospital (2023GJL202), and all experiments were conducted in accordance with relevant laws and institutional guidelines. Informed consent was obtained from all participants.

### 4.8. Statistical Analysis

Data are expressed as means ± standard deviation (*n* = 3). Analysis was performed using GraphPad Prism 8.3 software. The means of two groups were compared using Student’s *t*-test. Comparison of data from more than two groups was analyzed using one-way ANOVA (* *p* < 0.05, ** *p* < 0.01, *** *p* < 0.001, **** *p* < 0.0001).

## 5. Conclusions

In summary, this work establishes a robust colorimetric sensor without complex pretreatment for detecting ALP in clinical samples, leveraging the intrinsic matrix resistance of bimetallic AuPt nanozymes. Unlike conventional methods requiring complex sample pretreatment (e.g., deproteinization or ultrafiltration), our assay enables accurate quantification in human serum without any cleanup steps, achieving 95.45–111.97% recovery rates. The nanozyme’s exceptional tolerance to physiological interferents and high catalytic specificity underpin its reliability in complex biological matrices. With ultrahigh sensitivity (3.91 mU mL^−1^ LOD), rapid analysis (<30 min), and minimal operational requirements, this approach provides a practical paradigm for point-of-care ALP detection, demonstrating significant potential to transform clinical diagnostics through simplified sample processing workflows.

## Figures and Tables

**Table 1:** Comparison of the current work on colorimetric ALP determination.

Materials	Liner Range (mU mL^−1^)	LODs (mU mL^−1^)	Ref.
Fef NCs	0.2–40	0.19	[22]
ZnSA-AuAMP	0.47–10.03	0.09	[23]
IOP@Pt/Ru	0.1–100	0.05	[24]
Co-m-CeO_2_	3–72	0.021	[25]
Cu/CoS_2_	0.05–150	0.035	[26]
Au/T15/Pt	0.625–5.625	0.35	[27]
MIL-88B-NH_2_/Pt	1–50	1.89	[28]
Ru-C_3_N_4_	1.25–20	0.75	[29]
AuPt NPs	0–90	3.91	This work

**Table 2:** Recovery Analysis of ALP Activity in Serum Samples.

Sample	Added (mU mL^−1^)	Measured (mU mL^−1^)	RSD (100%)	Recovery (%)
1	0	0.80 ± 0.09	11.37	/
	10	9.69 ± 0.46	4.73	96.94 ± 4.59
	30	28.63 ± 1.75	6.10	95.45 ± 5.82
	60	60.31 ± 2.57	4.26	100.51 ± 4.28
2	0	0.60 ± 0.10	16.79	/
	10	10.78 ± 2.91	27.04	107.78 ± 29.14
	30	33.59 ± 3.55	10.57	111.97 ± 11.83
	60	60.78 ± 1.46	2.40	101.29 ± 2.44

## Data Availability

The datasets used in this study are available from the corresponding author upon reasonable request.

## Supplementary Materials

The following supporting information can be downloaded at: https://www.mdpi.com/article/10.3390/ph18121795/s1, Figure S1. Morphological characterization of AuPt NPs; Figure S2. Linear plot of absorbance at 652 nm versus AA concentration (A) and ALP concentration (B). (C) Selectivity test of AA detection. Table S1: Comparison of the Michaelis–Menten constant (*K*m) and maximum reaction rate (*V*max) of AuPt NPs with other catalysts. References [22,23,24,25,26,27,28,29] are cited in Supplementary Materials.
