# Supplementary material for: Synergistic Catalysis of Gold–Platinum Alloy Nanozymes: A Novel Colorimetric Sensor for ALP Detection in Complex Biological Matrices
Source: Pharmaceuticals (Basel). 2025 Nov 25;18(12):1795. doi: 10.3390/ph18121795 (PMC12735512; doi:10.3390/ph18121795)
Supplement: Supplementary file 1 [file pharmaceuticals-18-01795-s001.zip › pharmaceuticals-3970059-supplementary.pdf]

Supporting Information

Synergistic Catalysis of Gold–Platinum Alloy Nanozymes: A Novel Colorimetric Sensor for ALP Detection in Complex Biological Matrices

Baojie Du [1,†], Bingqing Zhang [2,†], Xiaofeng Ren [2], Jie Yang [1], Fan Yang [2], Chunyu Yan [2], Liping Li [3,*] and Ruiping Zhang [3,*]

[1] Shanxi Bethune Hospital, Shanxi Academy of Medical Sciences, Third Hospital of Shanxi Medical University, Tongji Shanxi Hospital, Taiyuan 030032, China; dubaojie@sxbqeh.com.cn (B.D.); yangjie@sxbqeh.com.cn (J.Y.)

[2] Department of Biochemistry and Molecular Biology, School of Basic Medical Sciences, Shanxi Medical University, Taiyuan 030001, China; b2024035015@student.pumc.edu.cn (B.Z.); dr20240041087@sxmu.edu.cn (X.R.); yangfan12@sxmu.edu.cn (F.Y.); yanchunyu@sxmu.edu.cn (C.Y.)

[3] Radiology Department of Shanxi Provincial People's Hospital, Five Hospital of Shanxi Medical University, Taiyuan 030001, China

* Correspondence: liliping@sxmu.edu.cn (L.L.); zrp_7142@sxmu.edu.cn (R.Z.)

† These authors contributed equally to this work.

Supplementary Figures

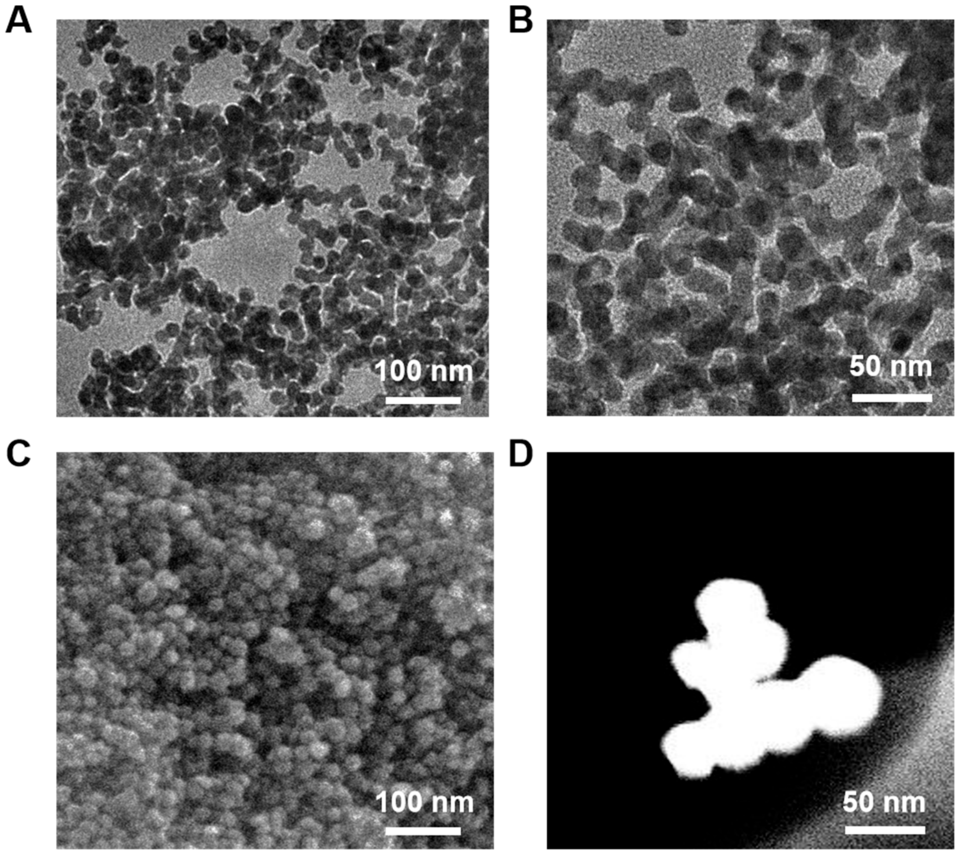

Figure S1. Morphological characterization of AuPt NPs. (A-B) Transmission electron microscopy (TEM), (C) Scanning electron microscopy (SEM), (D) Energy-dispersive X-ray spectroscopy (EDS) characterizations of AuPt NPs.

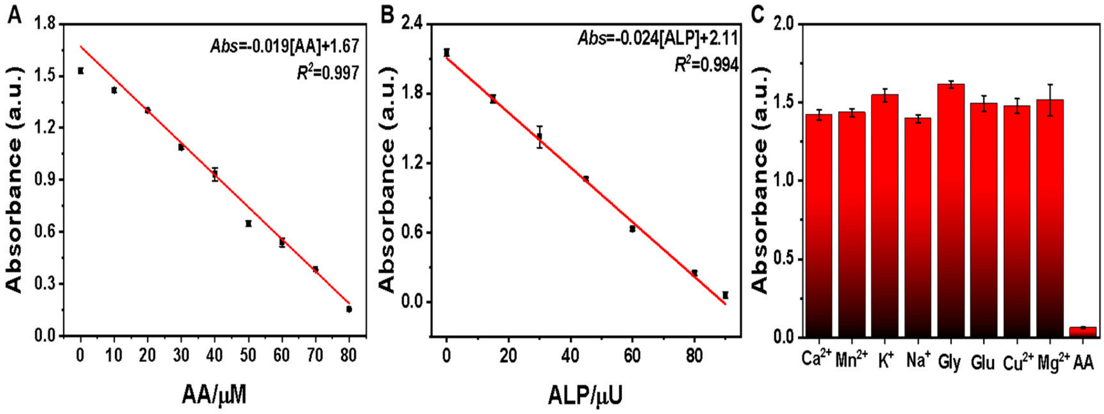

Figure S2. Linear plot of absorbance at 652 nm versus AA concentration (A) and ALP concentration (B). (C) Selectivity test of AA detection. The data indicate mean ± standard deviation. (error bars: repeated experiments; n = 3).

Table S1. Comparison of the Michaelis–Menten constant (K_m) and maximum reaction rate (V_{max}) of AuPt NPs with other catalysts.

Materials	Substrate	K_m (mM)	V_{max} (10^{-8} M s^{-1})	Ref
Fef NCs	TMB	0.22	19.6	[1]
	H_2O_2	0.01	9.34	
ZnSA-AuAMP	TMB	0.36	1.197	[2]
	H_2O_2	32.20	1.505	
IOP@Pt/Ru	TMB	1.3196	83.24	[3]
	H_2O_2	13.75	55.17	
Co-m-CeO_2	TMB	0.55	23.85	[4]
	H_2O_2	11.87	23.12	
Cu/CoS_2	TMB	2.70	19.4	[5]
	H_2O_2	0.079	3.0	
Au/T15/Pt	TMB	0.0417	1.377	[6]
	H_2O_2	27.071	0.144	
MIL-88B-NH_2/Pt	TMB	0.00213	1.025	[7]
	H_2O_2	0.026	2.0688	
Ru-C_3N_4	TMB	0.179	19	[8]
	H_2O_2	8.509	12.7	
AuPt NPs	TMB	0.050	12.79	This work
	H_2O_2	6.31	15.54	

References

1. Wu, H.; Bu, T.; Sun, B.; Xi, J.; Cao, Y.; Wang, Y.; Xuan, C.; Feng, Q.; Yan, H.; Wang, L. "Three-in-One" multifunctional hollow nanocages with colorimetric photothermal catalytic activity for enhancing sensitivity in biosensing. *Anal. Chem.* **2024**, *96*, 4825–4834.
2. Liu, L.; Jiang, H.; Wang, X. Alkaline phosphatase-responsive Zn(2+) double-triggered nucleotide capped gold nanoclusters/alginate hydrogel with recyclable nanozyme capability. *Biosens Bioelectron*. **2021**, *173*, 112786.
3. Gao, X.; Liu, L.; Hou, H.; Jia, W.; Zhang, A.; Zhang, B.; Bu, Y.; Gong, Y.; Yan, L.; Du, B. Construct a magnetic Pt/Ru alloy peroxidase mimic as a reusable and cost-effective "signal-off" sensing platform for sensitive and wide-linear-range assay. *Anal. Chem*. **2024**, *96*, 10467–10475.
4. Li, K.; Wang, J.; Wang, J.; Zheng, Z.; Liu, X.; Wang, J.; Zhang, C.; He, S.; Wei, H.; Yu, C.Y. A programmable microfluidic paper-based analytical device for simultaneous colorimetric and photothermal visual sensing of multiple enzyme activities. *Anal. Chem*. **2024**, *96*, 12181–12188.
5. Wang, H.; Su, P.; Wei, W.; Song, J.; Yang, Y. Hollow Cu/CoS(2) Nanozyme with defect-induced enzymatic catalytic sites and binding pockets for highly sensitive fluorescence detection of alkaline phosphatase. *Small*. **2024**, *20*, e2401416.
6. Cai, M.; Zhang, Y.; Cao, Z.; Lin, W.; Lu, N. DNA-programmed tuning of the growth and enzyme-like activity of a bimetallic nanozyme and its biosensing applications. *ACS Appl. Mater. Interfaces*. **2023**, *15*, 18620–18629.
7. Wang, M.; Zhao, Z.; Gong, W.; Zhang, M.; Lu, N. Modulating the biomimetic and fluorescence quenching activities of metal-organic framework/platinum nanoparticle composites and their applications in molecular biosensing. *ACS Appl. Mater. Interfaces*. **2022**, *14*, 21677–21686.
8. Ding, Z.; Li, Z.; Zhao, X.; Miao, Y.; Yuan, Z.; Jiang, Y.; Lu, Y. Self-deposited ultrasmall Ru nanoparticles on carbon nitride with high peroxidase-mimicking activity for the colorimetric detection of alkaline phosphatase. *Journal of Colloid and Interface Science*. **2023**, *631*, 86–95.
